# Quantitative Evaluation of Diffusion and Dynamic Contrast-Enhanced MR in Tumor Parenchyma and Peritumoral Area for Distinction of Brain Tumors

**DOI:** 10.1371/journal.pone.0138573

**Published:** 2015-09-18

**Authors:** Jing Zhao, Zhi-yun Yang, Bo-ning Luo, Jian-yong Yang, Jian-ping Chu

**Affiliations:** Department of Radiology, The First Affiliated Hospital, Sun Yat-Sen University, 58th, The Second Zhongshan Road, Guangzhou, Guangdong, China, 510080; University of North Carolina, UNITED STATES

## Abstract

**Purpose:**

To quantitatively evaluate the diagnostic efficiency of parameters from diffusion and dynamic contrast-enhanced MR which based on tumor parenchyma (TP) and peritumoral (PT) area in classification of brain tumors.

**Methods:**

45 patients (male: 23, female: 22; mean age: 46 y) were prospectively recruited and they underwent conventional, DCE-MR and DWI examination. With each tumor, 10–15 regions of interest (ROIs) were manually placed on TP and PT area. ADC and permeability parameters (K^trans^, Ve, Kep and iAUC) were calculated and their diagnostic efficiency was assessed.

**Results:**

In TP, all permeability parameters and ADC value could significantly discriminate Low- from High grade gliomas (HGG) (p<0.001); among theses parameters, Ve demonstrated the highest diagnostic power (iAUC: 0.79, cut-off point: 0.15); the most sensitive and specific index for gliomas grading were K^trans^ (84%) and Kep (89%). While, in PT area, only K^trans^ could help in gliomas grading (P = 0.009, cut-off point: 0.03 min^-1^). Moreover, in TP, mean Ve and iAUC of primary central nervous system lymphoma (PCNSL) and metastases were significantly higher than that in HGG (p<0.003). Further, in PT area, mean K^trans^ (p≤0.004) could discriminate PCNSL from HGG and ADC (p≤0.003) could differentiate metastases with HGG.

**Conclusions:**

Quantitative ADC and permeability parameters from Diffusion and DCE-MR in TP and PT area, especially DCE-MR, can aid in gliomas grading and brain tumors discrimination. Their combined application is strongly recommended in the differential diagnosis of these tumor entities.

## Introduction

Preoperative accurate brain tumor diagnosis plays an essential role in the selection of the optimum treatment strategy, as their management and prognosis are different. However, conventional structural imaging for accurate tumor diagnosis and grading is still challenging [1, 2]. Contrast enhancement on T1-weighted images reflects areas of blood brain barrier breakdown regardless of the pathology, which we used it to delineate tumor margin and grading of brain tumors. However, the enhanced tumor margin is not the real margin because of the infiltrative tumor cells can also be found in the peritumoral edema, especially in gliomas. And approximately 20% of low-grade gliomas enhance after administration of a gadolinium-based MR contrast agent, whereas approximately one third of nonenhancing gliomas are malignant [3, 4]; hence, the clinical application of conventional imaging in brain tumor diagnosis is limited and nonspecific.

Advanced MR imaging techniques, such as dynamic contrast enhanced MRI [DCE-MR] [5, 6] and diffusion weighted imaging (DWI) [7] can provide information on physiology and metabolism in vivo which is not available from conventional MR imaging that could be aid in gliomas grading, tumor margin definition and differential diagnosis of brain tumors. The diffusion and perfusion parameters are frequently used as independent imaging biomarkers for tumor grading and diagnosis. Previously, most of the studies were focused on capillary permeability measuring (K^trans^) and had already showed that K^trans^ could significantly differentiate brain tumor types and help gliomas grading [8– 10]. However, the grading and diagnostic ability of other permeability parameters (Ve, kep and iAUC) has not been well interpreted [11, 12]. Meanwhile, the utility of ADC in diagnosis of brain tumors and gliomas grading remains controversial. Lam WW [13] found ADC is useless for gliomas grading while the study of Kono K [14] and Kitis O [15] showed ADC could significantly differentiate brain tumors.

Further, most of investigators before were interested in the tumor parenchyma (TP) but not in the peritumoral (PT) area. While nearly all intra-axial brain tumors are accompanied with the PT area abnormality, which manifest as high signal intensity on T2-weighted MR imaging. From a pathological point of view, PT area signal abnormality represents a tumor induced increase in interstitial water. In high-grade gliomas (HGG) and primary central nervous system lymphoma (PCNSL), the PT area signal abnormality is not only caused by the altered interstitial water but also by the infiltration of the scattered tumor cells [16,17]. In contrast with the HGG and PCNSL, the infiltrated tumor cell could not be found in the PT area of metastases [18, 19]. Thus, inspecting PT area of the different brain tumor types might provide more meaningful information in brain tumor differentiation.

In the literatures most investigators focus on the TP but not on the PT area and only one parameter, K^trans^ was evaluated, but there is not enough comprehensive analysis of all other parameters from DCE-MR and DWI in different tumor area (TP and PT area) with respect to brain tumors differentiation. Further, the combination of DCE and DWI in brain tumor classification was seldom used in clinical application. Therefore, we present a comprehensive quantitative investigation of the diagnostic efficacy of DWI and DCE-MR parameters (K^trans^, Ve, Kep, iAUC and ADC), which acquired from TP and PT area in classification of brain tumors.

## Materials and Methods

### Patients

This study was approved by the research ethical committee of The First Affiliated Hospital of Sun Yat-Sen University according to ethical guidelines for human research and compliant with the Health Insurance Portability and Accountability Act (HIPAA). Written informed consent was obtained from adult patients or their legal guardians.

From November of 2012 to September of 2013, 71 consecutive patients with suspected brain tumor without enduring chemo-, steroid treatment and stereotactic biopsy were prospectively evaluated by conventional, DWI and DCE-MR. Inclusion criteria were: a) the diagnosis was confirmed by pathology except for meningioma that can be diagnosed precisely with clinical-radiological criteria (the tumor showed typical meningioma signal on MR [20], which was followed up for at least 1 year and the size and signal of the tumor had no obvious change); b) the PT area of each tumor was suitable for ROI placing (accordingly, the intraventricular tumors were excluded). Finally, 45 patients (male: 23, female: 22; mean age: 46 years) were included in our study, including 9 with low grade gliomas (LGG), 15 with HGG, 10 with meningiomas, 6 with PCNSL and 5 with metastatic tumors.

### MRI protocol and data post-processing

#### Conventional MR

Brain MRI was performed on all patients using a 3-T MR system (Magnetom Verio, Siemens Medical Solutions, Erlangen, Germany) and 12 phased-array brain coils. Transversal T2-weighted (TR 4000 ms, TE 100 ms, FOV: 230×230 mm; slice thickness: 5 mm; slice gap: 0.5 mm; voxel resolution: 0.7mm×0.6mm×6.0mm), transversal T1-weighted (TR 400 ms, TE 8.9 ms, FOV: 230×230 mm; slice thickness: 5 mm; slice gap: 0.5 mm; voxel resolution: 0.9mm×0.7mm×6.0mm) and coronary fluid-attenuated inversion recovery (TR/TE: 9000 ms/110 ms; inversion time, 2500 ms, FOV: 260×260 mm; slice thickness: 5mm; slice gap: 0.5mm; voxel resolution: 0.9mm×0.7mm×6.0mm) images were obtained. Postcontrast sagittal 3D T1-weighted (TR, 1880 ms; TE, 2.62 ms; section thickness, 1 mm; FOV, 256 ×256 mm; voxel resolution: 0.7mm×0.7mm×0.7mm) was obtained after DCE-MR.

#### Diffusion MR

DWI was performed by using an axial echo-planar spin-echo sequence (TR, 8700ms; TE, 88ms; slice thickness, 3 mm; FOV 260×260 mm; voxel resolution: 1.2mm×1.2mm×6.0mm). Diffusion was measured in 3 orthogonal directions by use of 3 b-values (0, 500, and 1000 s/mm^2^). ADC maps were automatically generated.

#### DCE-MRI

At the same slices of DWI, T1-VIBE was applied at two different flip angles (2° and 15°) to calculate the T1-maps. Below were the parameters (TR/TE, 5.21 ms/1.8 ms; slice thickness, 3mm; FOV, 210×210 mm; voxel resolution: 1.5mm×1.1mm×3.0mm). DCE-MRI was acquired with time resolved angiography with stochastic trajectories (TWIST) sequence, the parameters were following: TR/TE, 4.94 ms/1.93 ms; 10 slices; flip angle: 12°; slice thickness, 3mm; for each measurement, 3.6 s; FOV, 210×210 mm; 75 measurements, total scan time 276s; voxel resolution: 1.6mm×1.1mm×3.0mm; contrast media (0.1 mmol/kg body weight of Gd-DTPA, Magnevist, Schering, Berlin, Germany); contrast median injection rate: 3.5 ml/s for adults and 3ml/s for children, followed by 20ml of 0.9% saline flush using the same injection speed. Infusion started from the fifth measurement.

#### Data processing

All DCE-MRI data were transferred to post-processing workstation. Analysis was done by a commercial software tool (TISSUE 4D; Siemens Healthcare, Erlangen, Germany). A value for the arterial input function was automatically calculated using the software. TISSUE 4D was based on the two compartment model [21] and analyzed the data from three different angles: 1) three permeability parameters maps [volume transfer constant (K^trans^), extra-vascular extra-cellular volume fraction (Ve) and reflux constant (Kep)] were automatically generated with the fitted model and the mean value of each permeability parameter within the drawn ROI was recorded. 2) To quantitatively retrieve iAUC (initial Area Under Curve in first 60 seconds) and the mean value of iAUC in the drawn ROI was recorded as well. 3) Concentration-time curves (CTVs) of different ROIs in TP area were generated. According to the different manifestations of CTVs, we roughly divided them into three types: a) slowly rising; b) plateau; c) declining (slowly or quickly).

#### ROI placement

**Fig 1:**
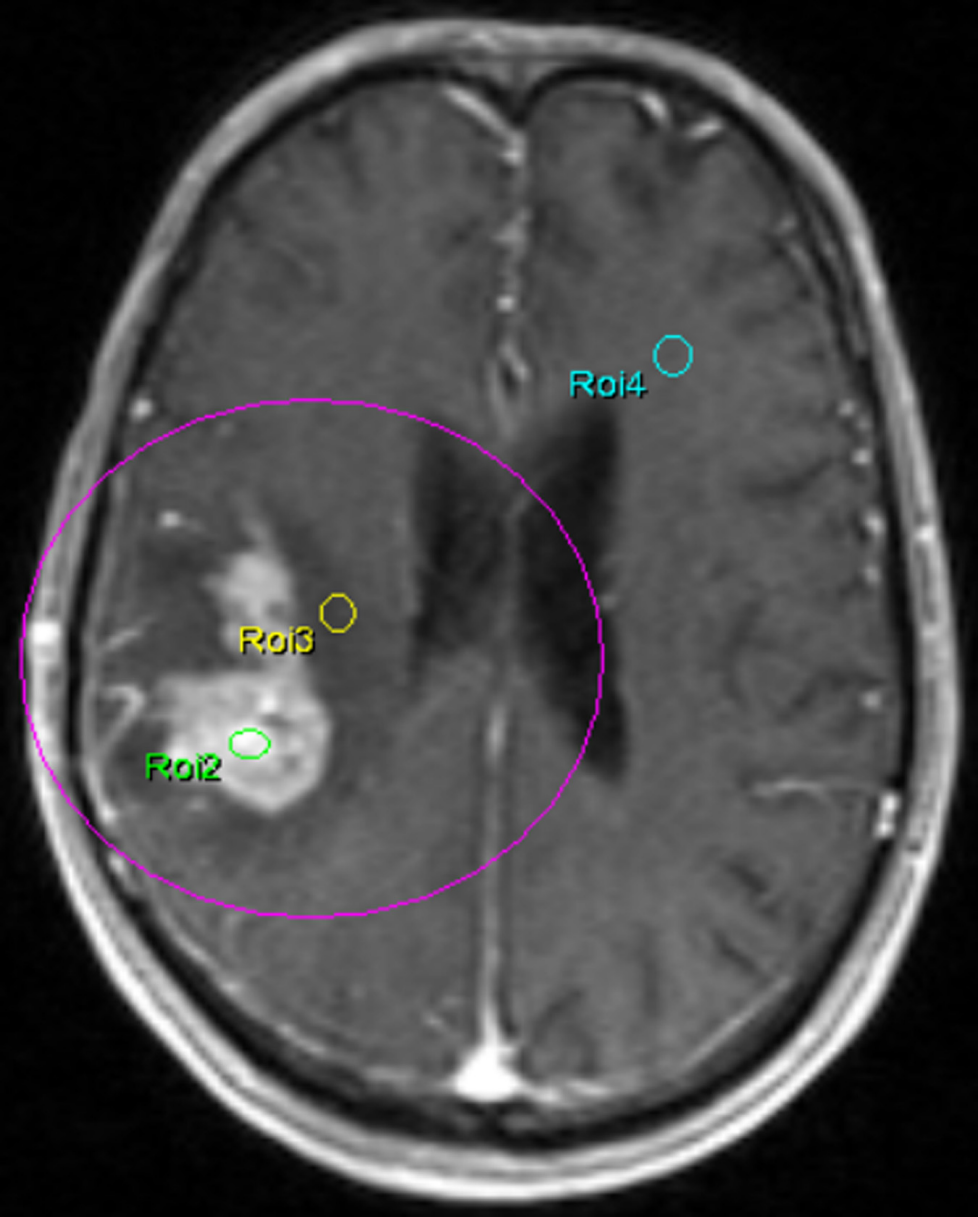
An example of ROIs placing. A 67 years old man with a suspicious high grade glioma in right temporal lobe on T1-weighted contrast-enhanced MRI. ROI 2 and ROI 3 were placed in the tumor parenchyma and peritumoral area, respectively; ROI 4 was put in the contralateral normal hemisphere.

ROI analysis was according to the recommendation of early studies [22–24]. ROI was placed after consensus of two experienced radiologists and the ROIs were placed within (TP) and around (PT) tumor area (within a 1cm distance from the outer enhancing tumor margin). The size of ROIs were similar to each other (0.25cm^2^–0.35cm^2^). 6–10 (depending on the size of the lesion) and 4–5 ROIs were separately placed in TP and PT area, according to enhanced T1w images, permeability parameter and ADC maps, avoiding the necrosis and cystic component, or, if the lesion did not show enhancement, such as LGG, T1 weighted and T2 weighted images were also needed for ROI placement. A ROI was placed in the normal contra-lateral hemisphere as well (Fig 1). Totally, 874 ROIs were placed in TP (permeability parameters maps: 450; ADC map: 424) and 447 ROIs were drawn on PT area (permeability parameters maps: 223; ADC map: 224). The detailed information of different ROIs with different types of brain tumor was summarized in Table 1.

**Table 1:** Statistic description (Mean and interquartile range) of the different brain tumor types in tumor parenchyma (TP) and peritumoral (PT) area.

ROIs	Index	LGG	HGG	Meningiomas	PCNSL	Metastase
		N = 9	N = 15	N = 10	N = 6	N = 5
**TP**	**K** ^**trans**^ **(min** ^**-1**^ **)**	**0.07 (0.02–0.11)**	**0.18 (0.10–0.23)**	**0.24 (0.14–0.31)**	**0.20 (0.08–0.32)**	**0.24 (0.15–0.30)**
	**Kep (min** ^**-1**^ **)**	**1.33 (0.77–1.52)**	**1.60 (0.45–1.02)**	**0.48 (0.31–0.62)**	**0.57 (0.43–0.74)**	**0.68 (0.44–0.93)**
	**Ve**	**0.10 (0.01–0.15)**	**0.27 (0.16–0.35)**	**0.49 (0.36–0.62)**	**0.38 (0.27–0.54)**	**0.37 (0.27–0.43)**
	**iAUC**	**4.40 (0.95–5.58)**	**10.32 (5.03–11.78)**	**21.87 (10.49–24.59)**	**13.16 (6.68–20.99)**	**15.84 (11.38–20.09)**
	**ADC (×10** ^**-3**^ **mm** ^**2**^ **/s)**	**1175.4 (1029.6–1397.3)**	**1048.5 (810.8–1266.6)**	**864.4 (762.4–958.7)**	**938.7 (742.3–1136.5)**	**1102.4 (864.6–1134.0)**
**PT**	**K** ^**trans**^ **(min** ^**-1**^ **)**	**0.03 (0.01–0.03)**	**0.04 (0.02–0.05)**	**0.03 (0.02–0.04)**	**0.03 (0.01–0.03)**	**0.02 (0.01–0.03)**
	**Kep (min** ^**-1**^ **)**	**3.14 (1.86–3.88)**	**4.11 (1.90–5.67)**	**2.39 (0.93–2.97)**	**2.82 (1.14–3.71)**	**3.05 (1.76–4.04)**
	**Ve**	**0.01 (0.01–0.02)**	**0.02 (0.01–0.03)**	**0.05 (0.02–0.04)**	**0.06 (0.005–0.02)**	**0.02 (0.01–0.03)**
	**iAUC**	**0.94 (0.63–1.12)**	**1.24 (0.34–1.80)**	**2.24 (1.08–2.66)**	**1.69 (0.39–1.24)**	**1.05 (0.35–1.74)**
	**ADC (×10** ^**-3**^ **mm** ^**2**^ **/s)**	**1292.9 (951.9–1586.2)**	**1398.5 (1156.8–1692.4)**	**1071.6 (757.1–1495.1)**	**1483.7 (1279.9–1752.6)**	**1645.7 (1525.0–1776.6)**

LGG: Low grade gliomas; HGG: High grade gliomas; PCNSL: primary central nervous system lymphoma

### Statistical analysis

Statistical analysis was carried out by SPSS (SPSS 16.0 Chicago, illinois). The association of the different quantitative permeability parameters and ADC value with tumor classification and gliomas grading were evaluated by analysis of Kruskal-Wallis. The relationship between different types of CTVs and tumor classification was evaluated by chi-square test. If the each of the global x^2^ was significant (p<0.05), Bonferroni analysis was used to assess difference between single groups, according to the corresponding multiplicity-adjusted P values. Receiver operating characteristic (ROC) analysis was performed for gliomas grading and tumors differentiation. Nonparametric estimates and 95% CIs for the area under the ROC curves (AUCs) were calculated. Optimal thresholds for gliomas grading were determined. Sensitivity, specificity, and the 95% CIs were calculated. P< 0.05 was considered statistically significant.

In order to further estimate the differential diagnostic effects of combining these parameters for tumor discrimination, several binary logistic regression models were constructed using the potential parameters as independent variables and the corresponding types of tumor that need to diagnosis as dependent variable. Then, ROC analysis using the predictive values from the logistic regressions were performed for tumor classification. The area under ROC curve with corresponding 95%CI was used to measure the capacity of the combination differential diagnosis. The optimal cut-off values of the continuous predictive values being transformed into binary variable was defined by a point on its ROC curve with maximum Youden index. The sensitivity and specificity of the optimal cut-off was reported.

## Results

### Gliomas Grading

Within TP, ROC curve analysis showed that all permeability parameters and ADC value could significantly differentiate HGG with LGG (Table 1). In TP, compared with LGG, K^trans^ (mean_LGG_ = 0.07 min^-1^ vs. mean_HGG_ = 0.18 min^-1^; P<.000), Ve (mean_LGG_ = 0.10 vs. mean_HGG_ = 0.27; P<.000), Kep (mean_LGG_ = 1.33 min^-1^ vs. mean_HGG_ = 1.60 min^-1^; P<.000) and iAUC (mean_LGG_ = 4.40 vs. mean_HGG_ = 10.32; P<.000) in HGG were significantly higher, while the mean ADC value (mean_LGG_ = 1175.4 mm^2^/s vs. mean_HGG_ = 1048.5 mm^2^/s; P = .001) were significantly lower; Ve demonstrated the highest diagnostic power (AUC: 0.79, 95%CI: 0.74–0.85), followed by K^trans^, iAUC, Kep and ADC. The sensitivity, specificity and cut-off point of Ve were 76%, 79% and 0.15. The most sensitive and specific index for gliomas grading were K^trans^ (84%, cut-off point: 0.07 min^-1^) and Kep (89%, cut-off point: 0.64 min^-1^), respectively.

The logistic regression analysis which included all mentioned significant parameters (Ve, K^trans^, Kep, iAUC and ADC) showed a higher discrimination power (p<0.001, AUC: 0.86) and better sensitivity (90%). The prediction value for HGG was calculated by the following equation (predictive value = 1.526*k^trans^+0.689*kep+23.571*ve-0.245*iAUC+0.0003*ADC-2.80) and the cut-off point was 0.45.

**Fig 2:**
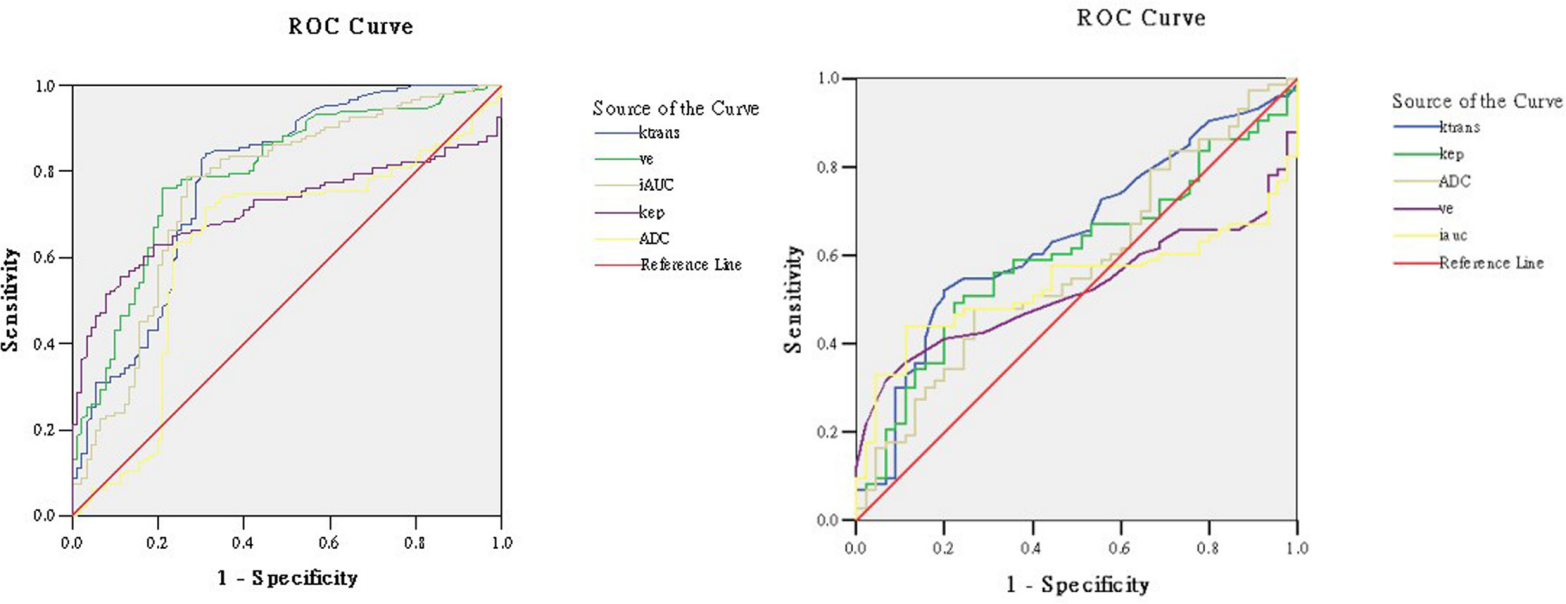
ROC curves for significant permeability parameters and ADC. Receiver operating characteristic (ROC) curves for significant permeability parameters and ADC in tumor parenchyma (Left) and peritumoral region (Right). In tumor parenchyma, Ve is the most powerful parameter to differentiate LGG from HGG, while in peritumoral area, only K^trans^ showed significant difference in low- and high grade glioma.

**Fig 3:**
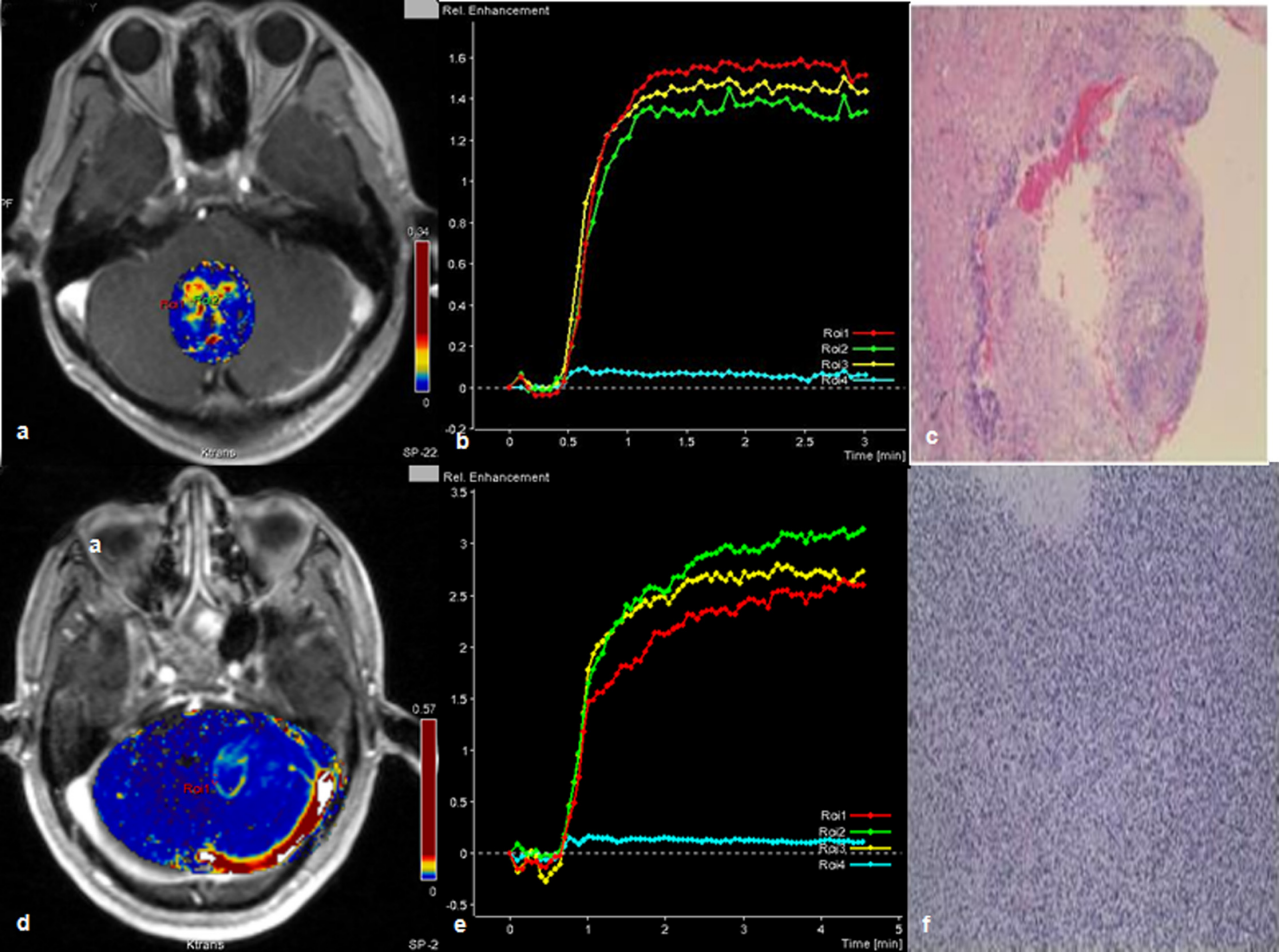
Comparison of HGG and LGG by k^trans^ and CTV. A 24 years-old male with low grade gliomas (WHO I) near the fourth ventricle (a, b, c) and a 59 years-old male with high grade gliomas (WHO IV) in the left cerebellar hemisphere(d, e, f). Permeability parameter (K^trans^) maps of the tumors (a,d); concentration-time curve (CTV) of each tumor: the CTV of the low grade glioma manifested as plateau (b), the other one showed as a slowly rising curve(e); specific histologic type of each tumor: pilocytic astrocytoma (c) (Hematoxylin- Eosin(HE) ×4), small cell glioblastoma (f), (HE ×10).

In PT area, ROC curve analysis displayed that only K^trans^ could differentiate HGG from LGG (mean_LGG_ = 0.03 min^-1^ vs. mean_HGG_ = 0.04 min^-1^; P = .009). The sensitivity, specificity and cut-off point of K^trans^ were 52%, 80% and 0.03 min^-1^ (Figs 2 and 3).

### Differential diagnosis of brain tumors

#### HGG vs. PCNSL

**Fig 4:**
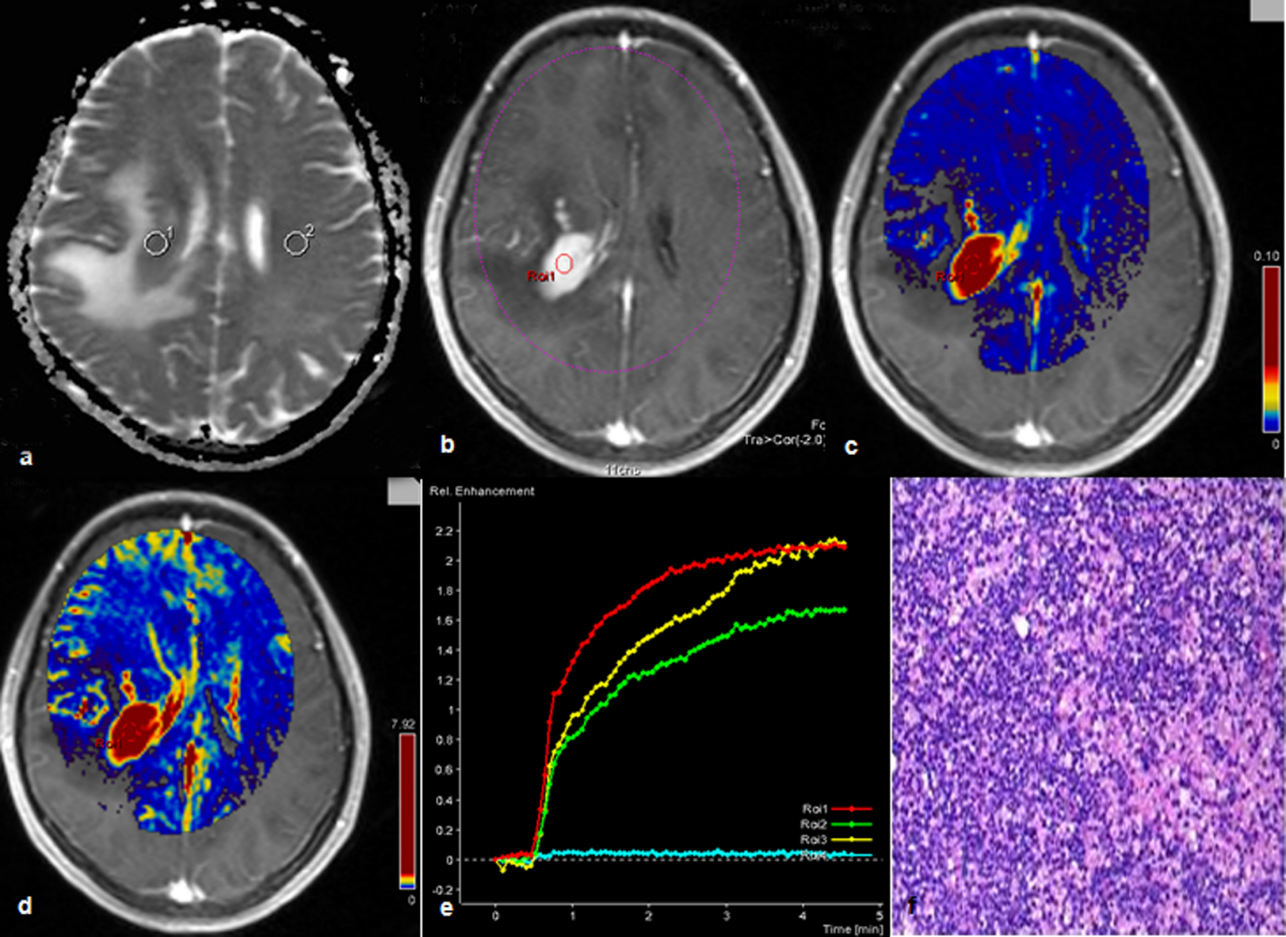
Description of primary central nervous system lymphoma by multiple parameters from diffusion and DCE-MR. A 16 years old female with primary central nervous system lymphoma in the right frontal lobe. ADC map showed that the tumor had decreased signal intensity with peritumoral edema (a); tumor on contrast-enhanced T1-weighted MRI manifested as severe enhancement (b); the corresponding Ve (c) and iAUC (d) maps showed higher value in tumor parenchyma than peritumoral area; concentration-time curve (CTV) of the tumor manifested as a slowly rising type (e); histologic results: large B cell lymphoma (f), (Hematoxylin- Eosin(HE) ×10).

Within TP, the mean values of Ve (mean_HGG_ = 0.27 vs. Mean_PCNSL_ = 0.38; p = .002) and iAUC (mean_HGG_ = 10.32 vs. Mean_PCNSL_ = 13.16; p = .003) in PCNSL (Fig 4) were significantly higher than HGG; Ve and iAUC demontrated a similar diagnostic power (AUC of VE: 0.64; AUC of iAUC: 0.63), but iAUC have a higher specificity (91%); In PT area, the mean values of K^trans^ (mean_HGG_ = 0.04 min^-1^ vs. Mean_PCNSL_ = 0.03 min^-1^; p = .004) were significantly lower than HGG; Interestingly, in TP area, the AUC of K^trans^ (0.68) is relatively higher than the AUC of Ve and iAUC. The sensitivity, specificity and cut-off point of K^trans^ were 78% and 60%, 0.0145 min^-1^, respectively.

#### HGG vs. Metastases

**Fig 5:**
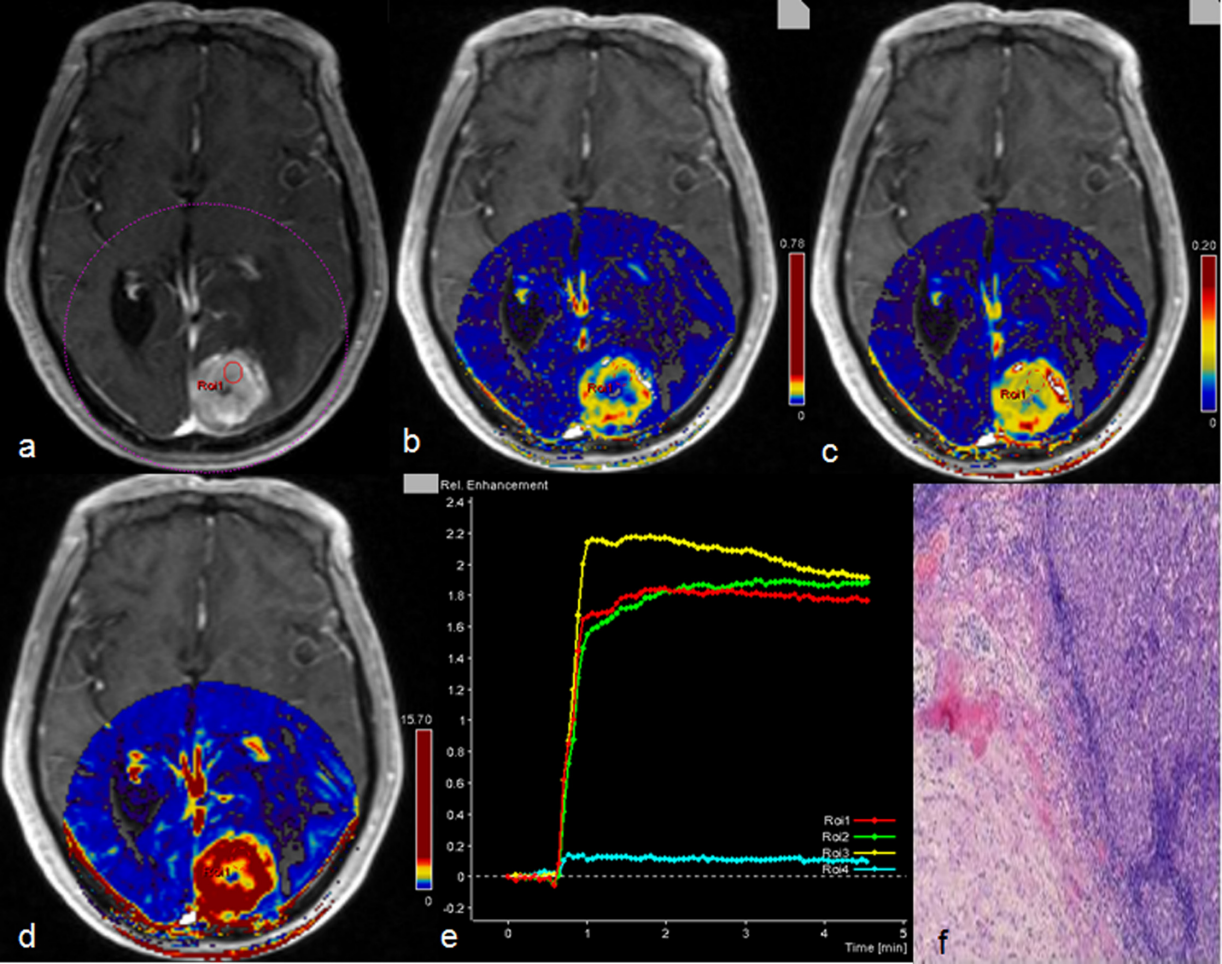
Description of metastatic tumor by multiple parameters from DCE-MR. A 72 years old male had a metastatic tumor in the left occipital lobe from renal cell carcinoma. Contrast-enhanced T1-weighted MRI showed that the tumor is obviously enhanced with severe peritumoral edema (a) and the corresponding K^trans^ (b), Ve (c) and iAUC (d) maps were also showed high value inside tumor parenchyma except central necrosis; concentration-time curve of the tumor (e), it manifested as plateau; brain metastases from renal cell carcinoma (f), (Hematoxylin- Eosin(HE) ×4).

Within TP, the mean values of K^trans^ (mean_HGG_ = 0.18 min^-1^ vs. Mean_Mets_ = 0.24 min^-1^; p<.000), Ve (mean_HGG_ = 0.27 vs. Mean_Mets_ = 0.37, p<.000) and iAUC (mean_HGG_ = 10.32 vs. Mean_Mets_ = 15.84; p<.000) of metastases (Fig 5) were significantly higher than HGG; The most powerful parameter for differentiation was iAUC (AUC:0.89), the sensitivity, specificity and cut-off point were 84%, 69% and 10.36, respectively. In PT area, ADC value (AUC:0.70; sensitivity: 96% and specificity: 47%) was useful in differentiating HGG with metastases, the mean value of ADC (mean_HGG_ = 1398.5 mm^2^/s vs. Mean_Mets_ = 1645.7 mm^2^/s; p = .003) in HGG was significantly lower than metastases.

#### Discrimination from LGG

Within TP, compared with LGG, the mean values of K^trans^, Ve and iAUC in meningiomas, PCNSL and metastases were significantly higher (p<.000), while the mean values of Kep and ADC (except in metastases) were obviously lower (p<.001); interestingly, in PT area, the mean value of ADC (mean_LGG_ = 1292.9 mm^2^/s vs. Mean_Mets_ = 1645.7 mm^2^/s; p<.003) in metastases was significantly higher (Table 1).

#### PCNSL vs. Metastases

Irrespective of ROIs in and around tumor area, there were no significant differences in permeability parameters and ADC values. However, combining analysis of Ve and iAUC in TP area by logistic regression analysis, we found that they could be used to differentiate PCNSL with metastases (p = 0.019) which have a mediate specificity (78%) but a relatively lower sensitivity (48%). The predicted value of metastases was calculated by the following equation (predicted value = -3.293*ve+0.116*iAUC-0.601).

Table 2 listed the parameters with the highest diagnostic prediction in gliomas grading and in the brain tumor differentiation.

**Table 2:** Parameters with the highest diagnostic prediction in tumor parenchyma (TP) and peritumoral area (PT) for distinction of a pair of brain tumors according to ROC analysis.

Comparison group	ROI Position	Most powerful parameter	AUC	Sensitivity	Specificity	Cut-off value
**HGG** ^&^ **vs LGG** ^$^	**TP**	**Ve**	**0.79**	**0.76**	**0.79**	**0.154** *
	**PT**	**K** ^**trans**^	**0.64**	**0.52**	**0.80**	**0.031min** ^**-1**^ *
**HGG** ^&^ **vs PCNSL** ^$^	**TP**	**Ve**	**0.64**	**0.53**	**0.79**	**0.389** ^#^
	**PT**	**K** ^**trans**^	**0.68**	**0.78**	**0.60**	**0.0145min** ^**-1**^ *
**HGG** ^&^ **vs Metastases** ^$^	**TP**	**iAUC**	**0.80**	**0.84**	**0.69**	**10.363** ^#^
	**PT**	**ADC**	**0.70**	**0.96**	**0.47**	**1357.4** ^#^
**Menigioma** ^&^ **vs LGG** ^$^	**TP**	**Ve**	**0.97**	**0.98**	**0.89**	**0.264** *
	**PT**	**ADC**	**0.65**	**0.67**	**0.70**	**1178.8** ^#^
**PCNSL** ^&^ **vs LGG** ^$^	**TP**	**Kep**	**0.87**	**0.76**	**0.77**	**0.818min** ^**-1**^ *
	**PT**	**ADC**	**0.65**	**0.63**	**0.69**	**1459.0×103mm** ^**2**^ **/s** ^#^
**Metastases** ^&^ **vs LGG** ^$^	**TP**	**Ve**	**0.93**	**0.82**	**1.00**	**0.196** *
	**PT**	**ADC**	**0.80**	**0.88**	**0.69**	**1471.6×103mm** ^**2**^ **/s** ^#^
**PCNSL** ^&^ **vs. Metastases** ^$^	**TP**	**No**	**-**	**-**	**-**	**-**
	**PT**	**No**	**-**	**-**	**-**	**-**

LGG: Low grade gliomas; HGG: High grade gliomas; PCNSL: primary central nervous system lymphoma

^&^: The first tumor group.

^$^: Comparative tumor group.

*: If the parameter’s value is higher than the cut-off point, the tumor is more likely to be the tumor type in the first tumor group.

^#^: If the parameter’s value is higher than the cut-off point, the tumor is more likely to be the tumor type belongs to the comparative tumor group.

### CTV

Detailed information of CTV with different brain tumor types was summarized in Table 3. Interestingly, the CTV of 60% patients with HGG were manifested as slowly rising type while only one (11%) LGG patient had slowly rising CTV (Figs 3, 4 and 5).

**Table 3:** Detailed information of concentration-time curves (CTVs) with different brain tumor types.

CTVs type	LGG		HGG		Meningiomas		PCNSL		Metastases	
	N	%	N	%	N	%	N	%	N	%
**Slowly rising**	**1**	**11%**	**9**	**60%**	**2**	**20%**	**1**	**17%**	**2**	**40%**
**Plateau**	**5**	**56%**	**5**	**33%**	**5**	**50%**	**4**	**67%**	**0**	**0%**
**Declining**	**3**	**33%**	**1**	**7%**	**3**	**30%**	**1**	**17%**	**3**	**60%**

LGG: Low grade gliomas; HGG: High grade gliomas; PCNSL: primary central nervous system lymphoma

## Discussion

Nowadays, new, sophisticated MRI techniques are being developed and applied in brain tumor diagnosis. DWI and DCE-MR are frequently used in clinical practice due to their relatively simple acquisition. Advanced, easy to read, post-processing software makes them even more popular. In order to fully explore and evaluate their ability in brain tumor diagnosis and new, well designed, prospective studies are necessary. In our study, based on conventional MR, we tried to analyze the utility of quantitative parameters from DCE-MR (k^trans^, Ve, Kep and iAUC) and DWI (ADC) in differentiation of brain tumors. Since meningiomas usually have typical imaging features on conventional MRI and could be easily diagnosed, more attention is given to glioma grading and other frequent brain tumor differential diagnosis, such as HGG vs PCNSL and HGG vs metastases. Our study demonstrated that quantitative ADC value, permeability parameters and measuring different ROIs in and around tumor area could help with the differential diagnosis.

### Gliomas grading

K^trans^ and Ve in TP area could significantly discriminate LGG from HGG. This result was concordant with previous researchers’s finding [8–10, 25]. Ve in our study demonstrated the highest discrimination power in gliomas grading, which was contrary with the previous studies and they found that K^trans^ had the highest diagnostic power [25, 26]; Further, barely no study before was focused on kep and iAUC, surprisingly, our study demonstrated that Kep had the highest diagnostic specificity in grading of gliomas. Tofts PS et al [27] found that K^trans^ was influenced by variable factors, such as microvascular blood flow, vessel permeability and vessel density. While Kep represented only vessel permeability and was not affected by blood flow; this maybe the reason why kep was more specific than k^trans^ to predict the grade of gliomas. In addition, contradicted with the results of Lam WW’s study [13], we did find that ADC value in HGG was significantly lower than LGG. As we know that HGG, compared with LGG, have a higher cellularity and ADC value is negatively correlated with tumor cellularity [14, 28–30]. Moreover, we combined all those parameters to perform an analysis, we found the diagnostic power had been well improved (AUC: 0.86).

Many researches had already demonstrated that HGG were more easily infiltrated their surrounding brain tissue and the relative cerebral blood volume (rCBV) around the tumors was significantly higher [22, 23, 31–33]. Similar to their results, our research was the first to show that K^trans^ in PT area of HGG was significantly higher than it in LGG. The diffuse tumor infiltration and associated neoangiogenesis of HGG may be the reason. Even though the sensitivity was relatively lower (52%), this result still inspired us to carry out more research of this kind in the future.

Further, we found that the CTVs of nine patients (60%) in HGG group manifested as slowly rising type while only one patient (11%) in LGG group with the similar curve type. However, the difference did not reach a significant point but may be due to our relatively small sample size. Compared with curve shape, iAUC values had more power in gliomas grading.

### HGG vs. PCNSL

Despite conventional MR characteristics were found to discriminate HGG from PCNSL. It was difficult, sometimes even impossible, to distinguish each other [34]. Our study discovered that iAUC in TP could be used to significantly differentiate the two types of tumor and the mean values of iAUC in PCNSL were significantly higher. The histological features of PCNSL (Infiltrated lymphatic cells build their network at the level of arterioles and venules [35–37]), which induces the severe enhancement could be used to explain our findings. On the other hand, compared with the highly vascularized HGG [38–40], the PCNSL lack of abundant neovascularization. This histologic difference of the tumor neovascularization could cause the different levels of K^trans^ values (as one of the most important measures of tumor vascular leakiness). However, in TP, we didn’t find any significant differences in K^trans^ and the mean values of K^trans^ in HGG even lower than in PCNSL. While, in PT area, the mean values of K^trans^ of HGG did significantly higher than that in PCNSL which was not only due to the different tumor neovascularization but also caused by the much more infiltrated growth manner of HGG.

We also found that the mean values of Ve of PCNSL in TP were obviously higher than that of HGG. Ve was positively correlated with the volume of trapped contrast agent in tumor interstitium [41]. The more contrast agent would be confined in tumor interstitium, the measuring Ve value was higher. A previous study showed that, in PCNSL, the contrast agent was restrained by a less pronounced disruption of the blood–brain barrier and/or by the perivascular lymphocytic cuffs [42], and this may explain PCNSL had a higher Ve value. In addition, with ADC, many studies had found, in TP area, it was useful in the differential diagnosis of HGG and PCNSL, because the cellularity of PCNSL is much higher than of HGG [15, 43–45]. However, our study failed to confirm it.

### HGG vs. Metastases

HGG and Intracranial metastases are two common brain tumors encountered in adults. In some instances, particularly when the lesion is solitary and clinical findings are noncontributory, conventional MRI alone cannot differentiate the two tumors. Many studies were carried on to investigate the efficiency of DWI and DCE-MR in differentiating each other. Moreover, due to the different growth manner of these two tumor types, the PT area was also a research hotspot. According to our results: in TP, the mean values of K^trans^, Ve and iAUC in metastases were significantly higher than the corresponding value in HGG and iAUC had the highest diagnostic power. To the limit of our knowledge, our study was the first to show these results which might due to the metastatic tumors in our study were hypervascularized, as the primary tumors were hepatic or renal carcinoma (n = 3); In PT area, most of the studies published before concluded that ADC value could be used for differentiation [33, 46], and our study also showed the similar results.

### PCNSL vs. Metastases

Our study didn’t show any significant quantitative DCE-MR and diffusion parameters in TP and PT were helpful in differentiation of PCNSL with metastases. However, the study of S. Wang et al [47] which included 16 PCNSL and 25 metastases inspected by DWI and dynamic susceptibility contrasted (DSC) MR showed that ADC could significantly differentiate PCNSL from metastases. Accordingly, the mean ADC of PCNSL in our cohort did lower than metastasis’s, but without reaching a significant level. Combining analysis of Ve and iAUC in TP area would help us to differentiate them, but the sensitivity is relatively low (48%).

Based on our results, we found that DCE-MR is more efficient in grading gliomas and differentiating the brain tumors. The limit of our study is the relative small sample size, a follow up study included more patients in this direction should be carried on.

## Conclusions

Advanced MRI techniques are required in many clinical cases where conventional MRI fails to differentiate malignant lesions such as HGG, PCNSL and metastases. DWI and DCE-MRI has been incorporated in the clinical routine to improve specificity and provide an insight into the underlying biological characteristics of brain tumors.

Our study demonstrated that the mean ADC, Kep, Ve and iAUC in TP and K^trans^ in both areas could significantly discriminate LGG from HGG. Among these parameters, Ve demonstrates the highest diagnostic power, while the most sensitive and specific parameter were K^trans^ and Kep, respectively. Combining analysis of all those parameters, the diagnostic power for gliomas grading had been well improved (AUC: 0.86). Moreover, the Ve and iAUC values in TP could be used to differentiate PCNSL or metastasis from HGG; while, in PT, K^trans^ and ADC were separately used in discrimination of HGG from PCNSL or metastases. Thus, quantitative ADC value and permeability parameters from DCE-MR and diffusion in TP and PT area, especially DCE-MR, can aid in gliomas grading and brain tumors differentiation. Their combined application is strongly recommended in the differential diagnosis of these tumor entities. We expect our results to contribute to perfusion analysis in diversity of clinical and research settings.

## Supporting Information

S1 Data Primary data of included brain tumors in tumor parenchyma. (XLS)
